# Palmatine Attenuates Metastatic Lung Colonization of Triple Negative Breast Cancer Cells

**DOI:** 10.3389/fphar.2022.853230

**Published:** 2022-04-13

**Authors:** Selase Ativui, Cynthia A. Danquah, Paul Poku Sampene Ossei, Michael Ofori

**Affiliations:** ^1^ Department of Pharmacology, Faculty of Pharmacy and Pharmaceutical Sciences, College of Health Sciences, Kwame Nkrumah University of Science and Technology, Kumasi, Ghana; ^2^ Department of Pathology, School of Medical Sciences, College of Health Sciences, Kwame Nkrumah University of Science and Technology, Kumasi, Ghana

**Keywords:** palmatine, triple negative breast cancer, lung metastasis, MTA1, p53

## Abstract

**Background:** Metastatic breast cancer to the lungs is a serious, life-threatening complication that is difficult to cure. Circulating tumor cells play a key role in the metastatic spread of breast cancer to the lungs *via* the lymphatic or circulatory system. Palmatine is a protoberberine alkaloid, identified as an active component of traditional African herbal preparations. Palmatine has antimetastatic and antiproliferative effects. The inhibitory activity of palmatine on the metastatic colonization of triple negative breast cancer cells in the lungs was investigated in this study.

**Methods:** 4T1 triple breast cancer cells were transplanted synergically to the thoracic duct of the female balb/c mice *via* the lymphatic system. Palmatine 1, 5 and 10 mg/kg were administered for 28 days. The lungs were analyzed for levels of arterial blood gas, histological damage, immunohistochemical expression of the metastasis-associated protein 1 (MTA1) and tumor suppressor p53 (p53).

**Results:** Administering palmatine 1–10 mg/kg dose dependently improved hypoxemia, ameliorated metastasis associated lung injury; histology score of 3.33 ± 0.33, 1.67 ± 0.33, 1.33 ± 0.33, decreased lung MTA1 (2.19 ± 0.12, 1.83 ± 0.04, 1.84 ± 0.05) and increased p53 expression (1.99 ± 0.06, 2.27 ± 0.12, 2.34 ± 0.12) respectively.

**Conclusion:** Palmatine preserved lung morphology and demonstrated therapeutic potential in aiding the treatment of lung metastasis.

## Introduction

Breast cancer is a disease frequently diagnosed among women marked by an abnormal growth of cells in the breast (Anjum et al., 2017). Lung metastasis occurs when breast cancer cells break away from the primary tumor and spread to the lungs. The tendency of breast cancer cells to form distant metastasis is a significant problem in its treatment (Medeiros and Allan, 2019). Circulating tumor cells contribute to the metastatic spread of breast cancer cells to the lungs. Circulating tumor cells are shed into the circulatory or lymphatics system from a primary breast tumor and function as seeds for the subsequent colonization and growth of breast cancer cells in the lung, a mechanism that is responsible for the vast majority of cancer-related mortality (Gupta and Massagué, 2006; Yang et al., 2019). Mouse models of metastasis have been instrumental in elucidating the underlying mechanisms of metastasis and tumor-host interactions required for colonization at distant sites. This assists in targeted interventions (Yang et al., 2012; Gómez-Cuadrado et al., 2017).

Despite progress in breast cancer therapy, once breast cancer cells develop in secondary organs the disease essentially enters an incurable phase (Leone et al., 2017). Efforts to identify novel therapeutic compounds that can aid the treatment of breast cancer metastasis are urgently needed.

Alkaloids from plants serve as a vital resource of novel drugs for anticancer therapy. Several alkaloids extracted from plants have shown *in vitro* and *in vivo* antiproliferative and antimetastatic properties against various forms of cancer. Notable examples include taxol analogs, vinca alkaloids and camptothecins currently used in cancer therapy (Lu et al., 2012; Choudhari et al., 2020).

Palmatine is a protoberberine alkaloid found in several medicinal plants including *Phellodendron amurense Rupr*. *(Rutaceae)*, *Coptis chinensis Franch. (Ranunculaceae)*, *Corydalis yanhusuo W.T.Wang ex Z.Y.Su & C.Y.Wu (Papaveraceae)*, *Tinospora cordifolia (Willd.) Hook.f. & Thomson (Menispermaceae), Tinospora sagittata (Oliv.) Gagnep*. *(Menispermaceae)* and *Stephania yunnanensis H.S.Lo (Menispermaceae)* (Long et al., 2019). *In vitro* investigations have demonstrated that palmatine exhibits anticancer and antimetastatic activities by inducing cell cycle arrest, apoptosis and inhibiting the migration/invasion of cancer cell lines (Hambright et al., 2015; Long et al., 2019). However, very few studies have investigated the antimetastatic activity of palmatine *in vivo*. This work investigated the inhibitory properties of palmatine on the metastatic colonization of breast cancer cells in the lungs and further explored the modulating activity of palmatine on a metastasis-related gene, the metastasis-associated protein 1 (MTA1).

## Materials

### Chemicals and Reagents

Palmatine (ChemShuttle, Trust Way, USA), Doxorubicin, Trypan blue (Sigma Aldrich, St. Louis, USA). Roswell Park Memorial Institute 1640 medium, Trypsin/EDTA, l-glutamine (Pan Biotech, Aidenbach, Germany), Fetal bovine serum, Penicillin-streptomycin (Capricorn scientific, Ebsdorfergrund, Germany), MTA1 (Cusabio, Houston, USA) and p53 Antibodies (Sigma Aldrich, St. Louis, USA).

### Cell Culture

The 4T1 triple negative breast cancer cell line (ATCC CRL-2539) was purchased from AddexBio (San Diego, CA 92117, USA). The 4T1 cells were cultured in Roswell Park Memorial Institute 1640 medium with 10% fetal bovine serum, penicillin 100 U/mL, streptomycin 100 g/ml and l-glutamine 2 mM. The cells were kept at 37°C in a 5% CO_2_ humidified atmosphere. Cell viability was determined with a trypan blue assay before the experiment.

### Animals

Female balb/c mice weighing 20–30 g and aged 6–8 weeks were purchased from the Noguchi Memorial Institute for Medical Research at the University of Ghana, Legon and kept at the animal house of the Department of Pharmacology, Kwame Nkrumah University of Science and Technology (KNUST), Kumasi. The mice were housed in stainless steel cages with delicate wood shavings as bedding, with free access to chow (GAFCO, Tema), and water. The room was maintained at a temperature of 24–28 C with a relative humidity of 60–70%, and a light-dark interval of 12 h. Before the experiment, the animals were acclimatized for a week.

## Methods

### Lung Metastasis

A fixed number of viable 4T1 triple negative breast cancer cells (2×10^6^) were transplanted *via* the lymphatic thoracic duct into each recipient mouse (Minn et al., 2005) after an anesthetisia of 50 mg/kg pentobarbitone *i.p*. was given. Following 48 h of inoculation, the animals were randomized into treatment groups;

### Animal Treatment Schedule

Animals were administered Palmatine (1, 5, 10 mg/kg *i.p*.), Doxorubicin (5 mg/kg, *i.p.* per week), Lung metastasis (4T1 triple negative breast cancer cells only, 2 × 10^6^) and Negative control (normal saline 1 ml/kg). After 28 days, the following assessments were made;

### Analysis of Arterial Blood Gases

Arterial blood was collected into heparinized tubes through a 23-gauge needle inserted into the aorta. Blood gas analysis was performed immediately after collection with a Blood gas and electrolyte analyzer BGE800 (Biobase, China).

### Histology

The lung tissues were excised and embedded in paraffin after being fixed in 10% neutral-buffered formalin. 4 µm thick deparaffinized sections were stained with hematoxylin and eosin (H&E) and analyzed at 10 × magnification. The histology sections were evaluated for metastatic lesions, morphological changes and necroinflammation according to a modified histology activity index (Gibson-Corley et al., 2013; Passmore et al., 2018). The sections were scored by a cumulative quantitative score of 0–4 representing; 0—Normal histological alterations, 1–3—Mild/moderate/severe and 4- Extreme histological alterations (Tables 1 and 2).

**TABLE 1:** Histology activity score for scoring lung sections.

Score	Organ Degeneration; pulmonary Features
0	None
1	Mild Inflammatory Exudate; areas of patchy Edema with some sisordered structure
3	Moderate Alveolar thickening (25–50% Visualized Lung)
4	Severe Alveolar Congestion (>50% visualized lung); Cellular debris filling air space; complete Loss of Alveolar Structure
—	Inflammation
0	No inflammation
1	Mild inflammatory exudate
3	Moderate inflammatory exudate
4	Severe inflammatory exudate
—	Fibrosis/lesions
0	No fibrosis
1	Minimal architectural distortion; Alveoli partly enlarged but no fibrotic masses
3	Fibrosis with architectural distortion but preserved lung structure
4	Lung structure severely damaged; Alveolar septa nonexistent with complete obliteration with fibrotic masses
Total score 12	—

**TABLE 2:** Cumulative histology activity score.

Score	Histology Score	Cumulative Score
0	Normal histological alterations	0
1	Mild histological alterations	1–3
2	Moderate histological alterations	4–6
3	Severe histological alterations	7–9
4	Extreme histological alterations	10–12

### Immunohistochemical Detection of MTA1 and p53 Expression

The immunoreactions of MTA1 and p53 were determined in the lung tissues with mouse polyclonal antibodies. In brief, 5 µm paraffin-embedded tissue sections were deparaffinized with xylene. The endogenous peroxidase activity was then quenched for 30 min in the dark with 3 percent H_2_O_2_ in methanol. Tissue sections were dehydrated in graded alcohols before performing heat-induced epitope antigen retrieval with 10 mM sodium citrate. Sections were washed with tris borate saline tween-20 (TBST) and then blocked for 1 h with 5 percent bovine serum albumin. The sections were incubated with the mouse polyclonal primary antibody. After washing for 5 min in TBST, the sections were incubated for 1 h with a horseradish peroxidase-conjugated rabbit anti-mouse IgG secondary antibody mixed with TBS in a 1:200 ratio. The sections were incubated with 3,3′-diaminobenzidine and counterstained with hematoxylin. Images of the slides were captured under a 10 × light microscope Leica DM2500 M (Leica Microsystems, Wentzler, Germany). The manifestation of a dark, brown-colored, intracytoplasmic precipitate is indicative of the presence of MTA1 and p53. The antibody stain intensity of the images obtained was analyzed with an IHC Profiler plugin in ImageJ. The Immunohistochemistry optical density score (IODS) was calculated (Seyed Jafari and Hunger, 2017).

$$\text{IHC optical density score}=\frac{\begin{array}{c} \text{Percentage}\;\text{contribution}\;\text{of}\;\text{high}\;\text{positive}\;\times 4+\text{Percentage}\;\text{contribution}\;\text{of}\;\text{positive}\;\times 3+\;\\ \text{Percentage}\;\text{contribution}\;\text{of}\;\text{low}\;\text{positive}\;\times 2+\;\text{Percentage}\;\text{contribution}\;\text{of}\;\text{negative}\;\times 1 \end{array}}{100}$$

### Statistical Analysis

Experimental data were expressed as Mean ± standard error of the mean. Analysis of data was done by Graph Prism software version 8.0.1- using One-way analysis of variance (ANOVA) followed by Dunnett’s post hoc tests. The criterion for significant difference between groups was set at *p* ≤ 0*.*05.

## Results

### Effect of Palmatine on Arterial Blood Gases

**FIGURE 1:**
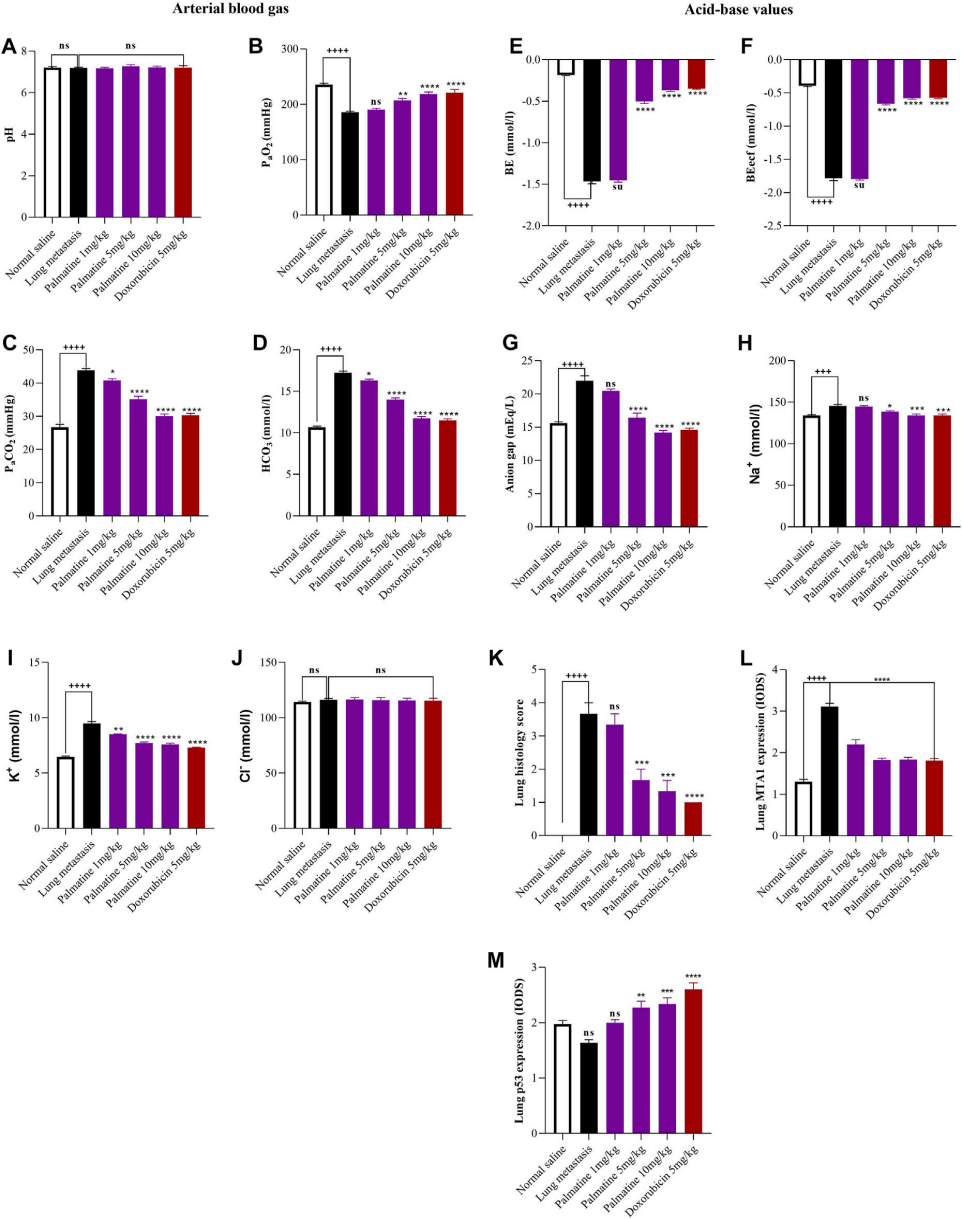
Effect of palmatine on Lung metastasis. Data represents mean ± SEM. *n* = 10, One-way analysis of variance followed by Dunnett’s post hoc test. Lung arterial blood gas: **(A)** potential of hydrogen (pH), **(B)** partial oxygen pressure (PaO_2_), **(C)** partial carbon dioxide pressure (PaCO_2_), **(D)** bicarbonate (HCO_3_). **(A)** ns (not significant) vs. Saline treated and Lung metastasis groups **(B)** ++++*p* < 0.0001 vs. Saline treated group and ns (not significant), **p = 0.002, ****p < 0.0001 vs. Lung metastasis group **(C)** ++++*p* < 0.0001 vs. Saline treated group and *p = 0.025, ****p < 0.0001 vs. Lung metastasis group **(D)** ++++*p* < 0.0001 vs. Saline treated group and *p = 0.0127, ****p < 0.0001 vs. Lung metastasis group. Lung acid-base values: **(E)** base excess (BE) **(F)** base excess extracellular fluid (BE ecf) **(G)** anion gap **(H)** sodium (Na^+^) **(I)** potassium (K^+^) **(J)** Chloride (Cl^−^). **(E–G)** ++++*p* < 0.0001 vs. Saline treated group and ns (not significant), ****p < 0.0001 vs. Lung metastasis group **(H)** +++*p* = 0.0002 vs. Saline treated group and ns (not significant), *p = 0.0112, ****p < 0.0002 vs. Lung metastasis group **(I)** ++++*p* < 0.0001 vs. Saline treated group and **p = 0.0016, ****p < 0.0001 vs. Lung metastasis group **(J)** ns (not significant) vs. Saline treated and Lung metastasis group. Lung immunohistochemistry: **(K)** lung histology scores **(L)** lung MTA1 expression **(M)** lung p53 expression. **(K)** ++++*p* < 0.0001 vs. Saline treated groups and ns (not significant), ***p = 0.001, ****p < 0.0001 vs. Lung metastasis group. **(L)** ++++*p* < 0.0001 vs. Saline treated groups and ****p < 0.0001 vs. Lung metastasis group. **(M)** ns (not significant) vs. Saline treated group and ns (not significant), **p = 0.002, ***p = 0.0007, ****p < 0.0001 vs. Lung metastasis group.

Palmatine (1–10 mg/kg) demonstrated a dose dependent increase in the partial oxygen pressure (P_a_O_2_) 190.70 ± 2.19, 207.0 ± 3.51, 218.5 ± 3.75 and decrease in the partial carbon dioxide pressure (P_a_CO_2_) 40.75 ± 0.58, 35.10 ± 0.92, 30.00 ± 0.68; bicarbonate (HCO_3_) 16.30 ± 0.17, 14.00 ± 0.20, 11.75 ± 0.20; base excess (BE) −1.45 ± 0.02, −0.50 ± 0.03, −0.37 ± 0.01 and anion gap 20.46 ± 0.27, 16.41 ± 0.70, 14.19 ± 0.32 respectively compared to a decreased PaO_2_ 185.70 ± 1.89, increased PaCO_2_ 43.83 ± 0.51; HCO_3_ 17.23 ± 0.20, BE −1.47 ± 0.02 and anion gap 21.98 ± 0.78 in the untreated lung metastasis group consistent with hypoxemia and respiratory distress. This resulted in reduced activity levels and labored breathing. There were no significant changes in values recorded for potential of hydrogen (pH) and chloride (Cl^−^) for all groups in the experiment (Figure 1A–J)

### Effect of Palmatine on Lung Histology

**FIGURE 2:**
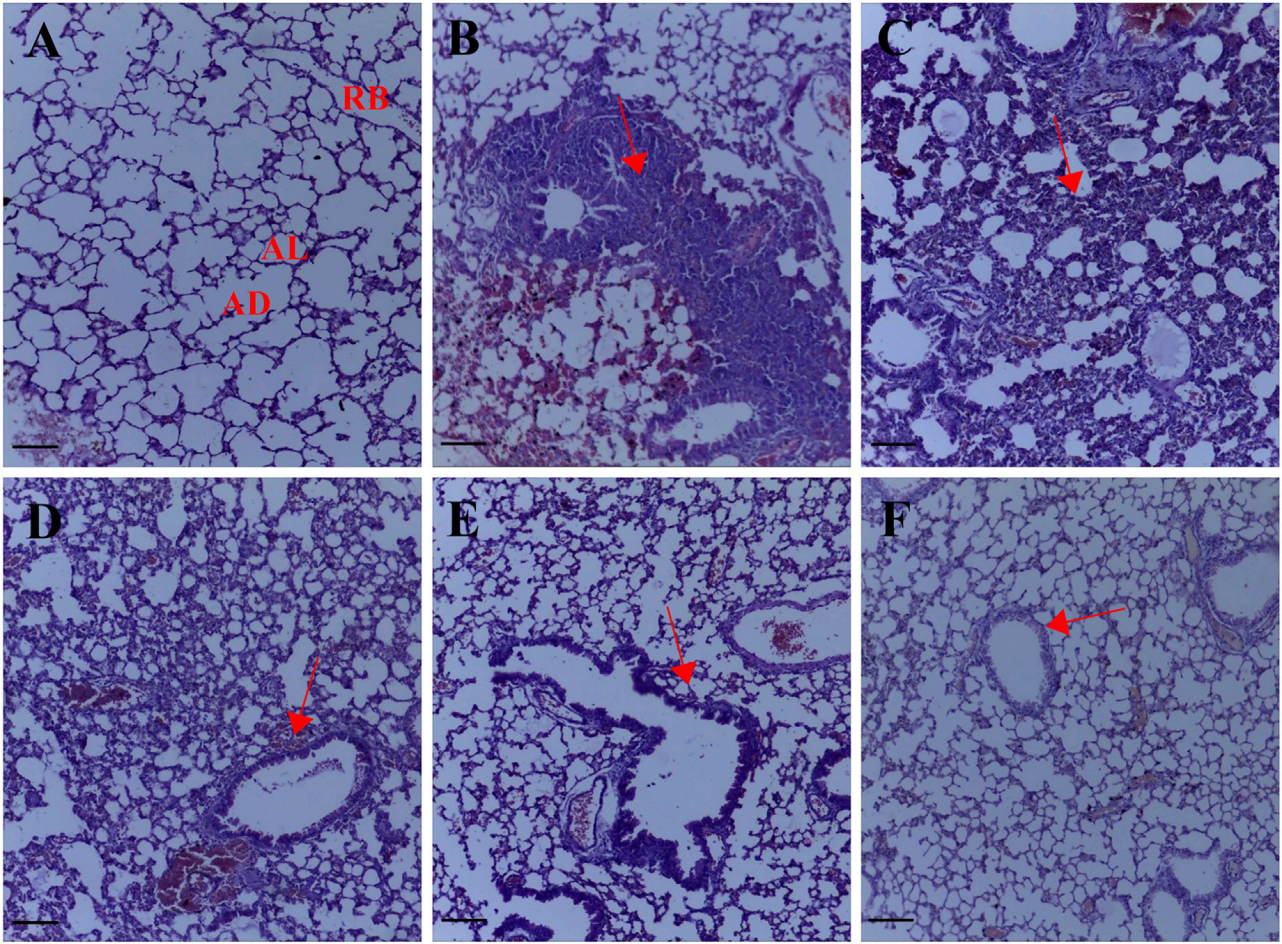
Representative photomicrographs of the lung H& E sections. **(A)** Normal saline **(B)** lung metastasis **(C–E)** Palmatine 1–10 mg/kg **(F)** Doxorubicin 5 mg/kg; AL, alveolus; AD, alveolar duct; RB, respiratory bronchiole. Red arrows show metastatic alterations. Scale bar 20 mm ×10, **(A–F)**.

After transplantation of the 4T1 breast cancer cells, metastatic colonization resulted in a remodeling of the lung parenchyma, a large metastatic growth of breast cancer cells marked by peribronchial and perivascular inflammation with the formation of lymphoid nodules. A histology score of 3.67 ± 0.33 (Figures 2B, 1K) was obtained for the untreated lung metastasis group. Palmatine 1 mg/kg showed diffused interstitial fibrotic changes, thickening of alveoli epithelial cells typical of metastatic progression. Administering palmatine 5 and 10 mg/kg dose dependently preserved lung morphology with increased interstitial spaces, thin-walled alveoli and attenuated metastasis-associated endothelial injury; a histology score of 3.33 ± 0.33, 1.67 ± 0.33, 1.33 ± 0.33 (Figures 2C–E, 1K) was obtained for palmatine 1–10 mg/kg respectively.

### Immunohistochemical Detection of MTA1 Expression

**FIGURE 3:**
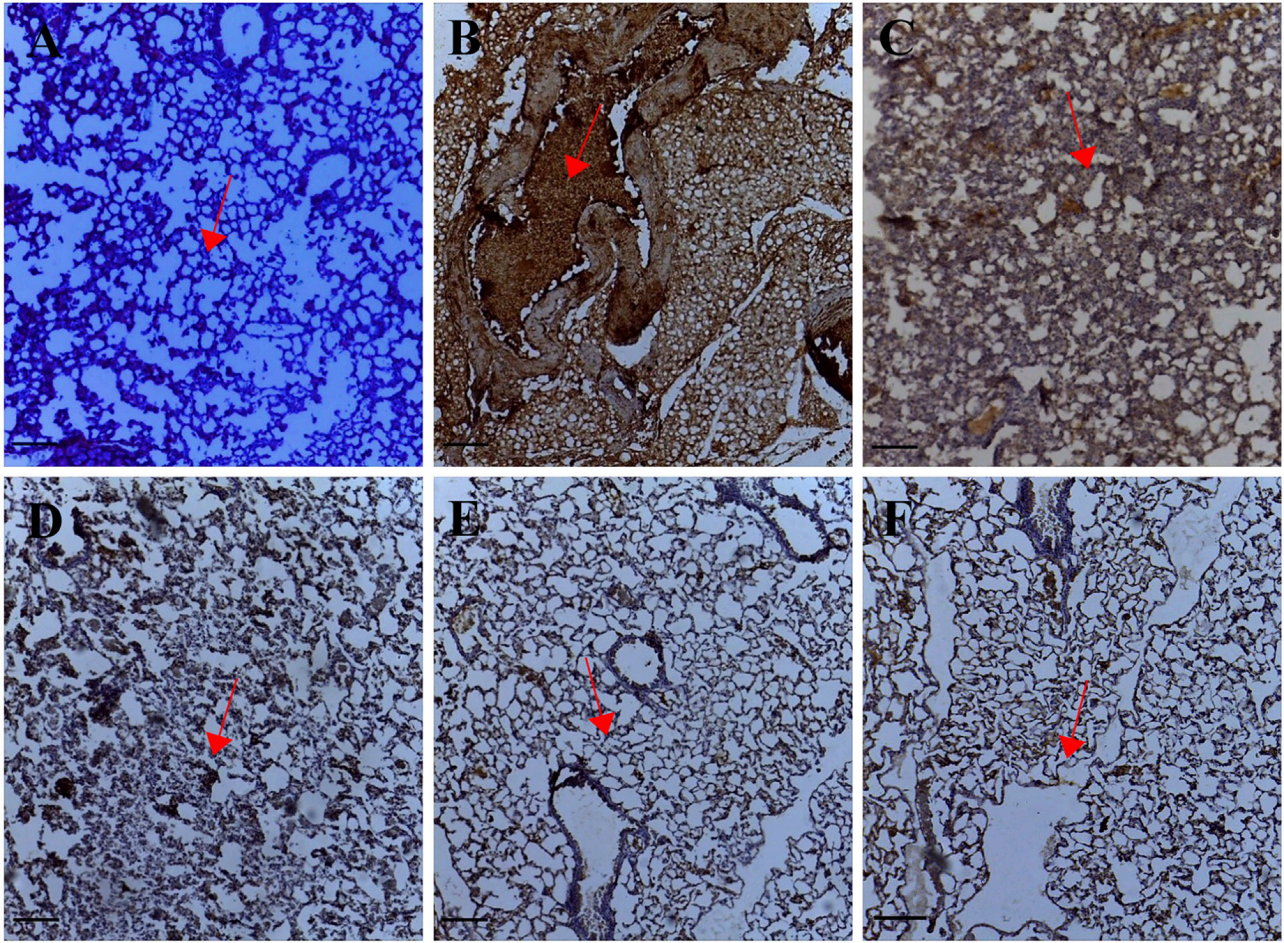
Representative photomicrographs of MTA1 expression in IHC sections of the lung tissue: **(A)** Normal saline **(B)** Lung metastasis **(C–E)** Palmatine 1–10 mg/kg **(F)** Doxorubicin 5 mg/kg. Data were obtained from five fields per slide with seven slides analyzed from each group. Red arrows show immunoreactive areas. Scale bar 20 mm (×10, **(A–F)**.

The expression of MTA1 indicated by a brown stain was evaluated by a semi-quantitative method using the Immunohistochemistry Optical density score (IODS). Tissue sections from the untreated lung metastasis group showed positive immunoreactivity in the cytoplasm for the MTA1 protein; IODS 3.11 ± 0.07 (Figures 3B, 1L) compared to the low expression in palmatine treated groups 1–10 mg/kg; IODS 2.19 ± 0.12, 1.83 ± 0.04, 1.84 ± 0.05 respectively (Figures 3C–E, 1L).

### Immunohistochemical Detection of p53 Expression

**FIGURE 4:**
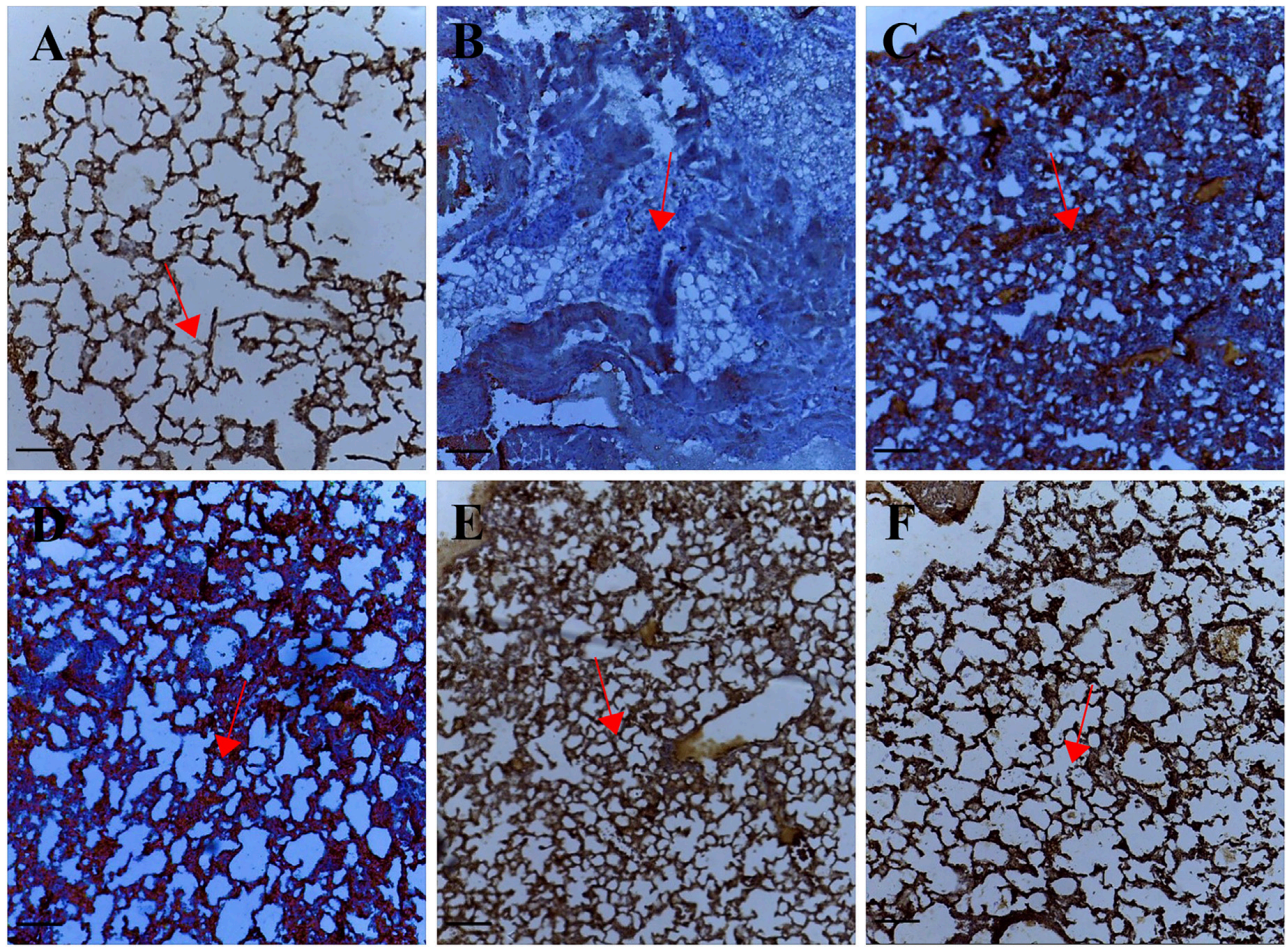
Representative photomicrographs of p53 expression in IHC sections of the lung tissue: **(A)** Normal saline **(B)** Lung metastasis **(C–E)** Palmatine 1–10 mg/kg **(F)** Doxorubicin 5 mg/kg. Data were obtained from five fields per slide with seven slides analyzed from each group. Red arrows show immunoreactive areas. Scale bar 20 mm (×10, **(A–F)**.

Tissue sections from palmatine treated groups 1–10 mg/kg showed positive immunoreactivity in the cytoplasm for the p53 protein; IODS 1.99 ± 0.06, 2.27 ± 0.12, 2.34 ± 0.12 respectively (Figures 4C–E, 1M) compared to the low expression in the untreated lung metastasis group; IODS 1.64 ± 0.06 (Figures 4B, 1M).

## Discussion

From the “seed and soil hypothesis”, colonization of organs requires complex adaptations by cancer cells to various host components. Intercommunication between cells is steered by a dynamic network of cytokines, chemokines, growth factors, inflammatory and matrix remodeling enzymes against a background of major perturbations to the physical and physiological functions of the tissue (Whiteside, 2008; Massagué and Obenauf, 2016).

Breast cancer cells interact with the lung stroma such as the mesenchymal stem cells, fibroblasts and myeloid cells (including monocytes, macrophages and neutrophils) through several paracrine signaling cascades that allow the formation of immunotolerant niches to promote cancer cell proliferation (Celià-Terrassa and Kang, 2018; Altorki et al., 2019). To a significant extent, metastatic colonization resembles chronic lung inflammation with extensive remodeling of the extracellular matrix (Li et al., 2017).

Evidence from our previous investigation showed that palmatine could protect against triple negative mammary carcinoma in female balb/c mice which was associated with the activation of the endogenous tumor suppressor gene p53 (Ativui et al., 2021). *In vitro* experiments have also demonstrated the efficacy of palmatine against tumor migration and invasion (Hambright et al., 2015; Qi et al., 2019). This research was conducted to validate the efficacy of palmatine in protecting breast cancer metastasis *in vivo*.

To investigate the effects of palmatine on metastatic lung colonization, an arterial blood gas test (ABG) was performed. The ABG test analyzed the arterial partial pressure of oxygen (PaO_2_), the arterial partial pressure of carbon dioxide (PaCO_2_), and acid-base disturbances. An ABG test assesses gas exchange through the alveolar-capillary membrane and lung function. Such knowledge is important to evaluate the severity of respiratory disease in lung metastasis. Mice in the untreated lung metastasis group showed high levels of PaCO_2_ while PaO_2_ was low with impaired acid-base homeostasis consistent with hypoxemia and underventilation from impaired lung function. These observations were however improved in palmatine-treated mice.

The formation of lung metastases constitutes a variety of genes and pathways. The Metastasis associated protein 1 (MTA1) is a member of the nuclear remodeling histone deacetylate complex, a critical modulator of chromatin remodeling. The MTA1 gene is upregulated in several cancers (Simpson et al., 2001; Pakala et al., 2013). Overexpression of MTA1 has been linked to the promotion of metastasis in animal tumor models and human cohort studies by facilitating the activation of epithelial-mesenchymal transitions, expression of angiogenic factors, cancer cell proliferation, aggressive phenotypes, and invasion. In addition, MTA1 enhances metastasis signaling pathways such as the Wingless-related integration/E-cadherin/-catenin, hedgehog, notch, cell cycle regulatory, and growth factor receptor signaling (Ghanta et al., 2011; Sen et al., 2014). The transcription of MTA1 is inhibited by the tumor protein p53 (Li et al., 2009). These documented roles of MTA1 make it a valuable target to be explored in the metastatic colonization of breast cancer cells. Subsequently, administering palmatine inhibited the expression of MTA1 while lung expression of p53 was increased preventing metastatic alterations to the lung architecture.

## Conclusion

Palmatine greatly preserved lung morphology and restored respiratory function demonstrating therapeutic potential in attenuating metastatic colonization of breast cancer cells in the lungs.

## Data Availability

The original contributions presented in the study are included in the article/Supplementary Material, further inquiries can be directed to the corresponding author.
